# Experimental infection of a periodical cicada (*Magicicada cassinii*) with a parasitoid (*Emblemasoma auditrix*) of a proto-periodical cicada (*Okanagana rimosa*)

**DOI:** 10.1186/s12898-014-0031-7

**Published:** 2014-12-14

**Authors:** Reinhard Lakes-Harlan, Thomas de Vries

**Affiliations:** Integrative Sensory Physiology, Institute of Animal Physiology, Justus-Liebig-University Giessen, Heinrich-Buff Ring 26, D 35392 Giessen, Germany

**Keywords:** Host location, Host suitability, Evolution of periodicity, Auditory system

## Abstract

**Background:**

The proto-periodical cicada *Okanagana rimosa* is subject to infection by the acoustically orientating parasitoid fly *Emblemasoma auditrix.* Furthermore, it is also the only known host of *E. auditrix*. Here we test the question, whether the highly adapted parasitoid can also infect other cicadas, like the periodical cicada *(Magicicada cassinii)* and which steps of the parasitization process can be completed. The experiments might also reveal whether such a parasitoid could hypothetically have been involved in the evolution of periodicity.

**Results:**

The hearing threshold of *E. auditrix* matches with the spectrum of the calling song of *M. cassinii*, indicating potential host localization. Behaviourally, host localization is possible by the parasitoid as it approaches a loudspeaker broadcasting the buzz part of the calling song of *M. cassinii. Magicicada cassinii* is readily accepted as host and for host infection the parasitoid uses the same behavioural sequence as for its host *O. rimosa*. A larva is deposited into the timbal of the cicada. By contrast to *O. rimosa* the development of the fly larva is delayed and eventually suppressed in *M. cassinii*.

**Conclusions:**

The host range of *E. auditrix* is mainly determined by acoustic parameters. This filter is important, as other sensory cues seem not to be involved in the host selection process and larva will not develop in unsuited host. Although the recent parasitoid-host system seems to be stable in terms of coexistence of both species, an acoustically hunting parasitoid could have been a selective force during evolution of prime numbered periodicity in cicadas.

## Background

Adults of the three different species of periodical cicadas, *Magicicada cassinii* (Fisher), *M. septendecim* (Linnaeus) and *M. septendecula* (Alexander & Moore), appear every 17 years above ground [1]. Their nymphs live underground where they feed on roots before emerging as adults. The unarmed and harmless insects are attacked by parasitoids and predators, like birds and mammals, often until the predators are satiated [1]. The survival of these cicada populations is probably secured by their high abundance and by their unusual life cycle [1,2]. The evolutionary origin of this long periodic life cycle with synchronous emergence of three reproductively isolated species is subject to discussion for a long time [1,3]. Model calculations have shown that such a periodic life cycle with prime numbers can be a result of the selective pressure of parasites [4]. However, the model is based on hypothetical parasites. Therefore we experimentally tested whether the periodical cicada *Magicicada cassinii* could be a potential host of the geographically separated parasitoid fly *Emblemasoma auditrix* (Soper).

Females of the parasitoid fly *Emblemasoma auditrix* (Diptera, Sarcophagidae) acoustically locate their host, the male cicada *Okanagana rimosa* (Say) (Hemiptera, Cicadidae) [5,6] and distributions of both species overlap at least in the South of Ontario (Canada) and Michigan (USA). The auditory cue is the calling song of the male cicada intended to attract conspecific females [7]. *Emblemasoma auditrix* has evolved a tympanal organ at the prothorax to overhear that song and to localize the cicada [8]. When a fly finds a host, it approaches the host and rapidly deposits a fully developed larva into the timbal of the cicada [9]. The larva feeds inside the cicada and leaves it after about six days thereby killing the host [9]. *Okanagana rimosa* is so far the only known host of *E. auditrix* and it remains to be shown which parameter limits the host range of the parasitoid.

Therefore we tested with the experimental infection of periodical cicadas also the limits and effectiveness of the parasitization process. We performed experiments to analyse three steps of the parasitization process: host location, host acceptance and host suitability [10]. Although *M. cassinii* seems not to be a suitable host in our experiments, the initially successful parasitization process suggests that this recent parasitoid might serve as a factor in the model for the origin of periodicity and species separation in cicadas.

## Results

**Figure 1:**
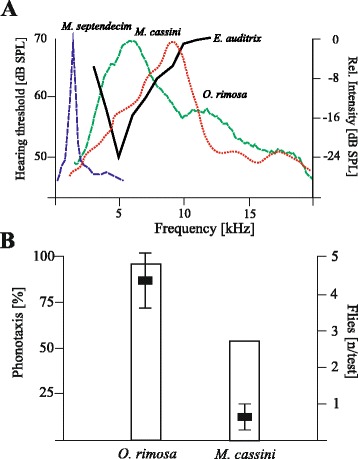
**Hearing threshold and phonotactic behaviour of** ***E. auditrix*** **. A** The hearing threshold of *E. auditrix* is depicted as a solid black line (modified after [8]). The calling song spectra have been analyzed by Fast Fourier Transformation: host cicada *O. rimosa* (dotted red line), periodical cicadas *M. cassinii* and *M. septendecim* (stippeld green and blue lines). Note the overlap of hearing range with the spectrum of *M. cassinii,* whereas *M. septendecim* can hardly be detected acoustically. **B** Phonotaxis of *E. auditrix* in behavioural tests in the laboratory (columns; left y-axis) and in attraction experiments in the field (black squares; mean, s.e.m.; right axis). In the laboratory arena phonotaxis was tested towards calling songs (80 dB SPL at 50 cm); the arrival at the loudspeaker is plotted as percentage of the number of animals (22 replicates for each song). In the field, flies were counted which arrived at the loudspeaker during a three minute broadcast of the respective song model (100 dB SPL at 1 m distance) (5 replicates for each model).

The parasitoid *E. auditrix* uses the acoustic signal of the cicada *O. rimosa* as long range cue for host location. Therefore, we first correlated the hearing range to the frequency content of the calling songs of cicada species. The hearing range of the fly and the calling song spectrum of the periodical cicada *M. cassinii* match in the range of 4–7 kHz (Figure 1A). This overlap enables phonotaxis of *E. auditrix* to the calling song of *M. cassinii* and is even more pronounced than that in the recent parasitoid-host system (*E. auditrix - O. rimosa*). The hearing threshold does not match to the spectrum of *M. septendecim*.

Secondly, phonotaxis tests have been performed. In the field almost no fly could be attracted to the calling songs of *M. cassinii* or *M. septendecim*. However, flies did respond to song models of the buzz part of the calling song of *M. cassinii* (Figure 1B). Nevertheless, the number of attracted flies was lower than those to the song model of the host (Figure 1B). In behavioural tests in the laboratory almost all flies (96%; n = 22) reacted with an initial turn towards the signals of both, *O. rimosa* and *M. cassinii.* The percentage of flies continuing and completing phonotactic approach depended on the signal (Figure 1B), with the calling song of *O. rimosa* being the more attractive signal. Nevertheless, 55% of flies completed phonotaxis towards the signal of *M. cassinii* (Figure 1B).

**Figure 2:**
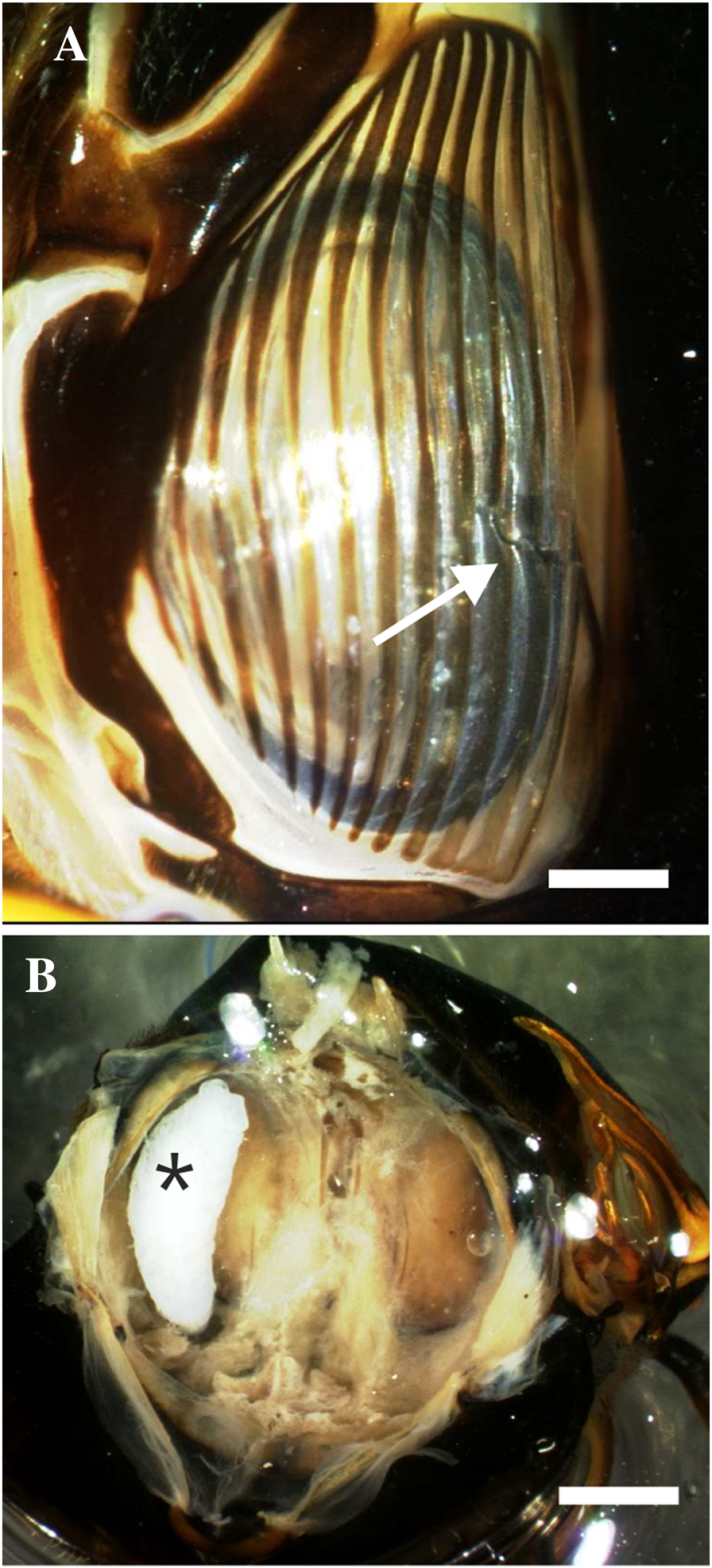
**Photomicrographs of the injured timbal and the larva feeding inside the host cicada. A** Timbal of *M. cassinii* with an injury at the ribs 8–11 (arrow). The ribs were broken during the injection of the parasitoid larva. **B** Second instar larva (asterisk) lying in the abdomen close to the timbal cavity of a host cicada. Scales: A: 500 μm; B: 2 mm.

For experiments on the second step of parasitization, host acceptance, male *M. cassinii* were restrained to a loudspeaker and presented to acoustically attracted flies. *Emblemasoma auditrix* landed on or nearby the cicada, moved around the potential host, visually identified the abdomen and approached the host from the caudal side. The parasitoid squeezed underneath the wings and deposited one larva into the timbal, the sound producing organ of the cicada. The timbal was usually injured posterior-ventrally at the timbal ribs 8–11 (Figure 2A) and 2 to 3 ribs (mean 2.4; s.e.m. 0.12; n = 40) were broken. During experimental infection *M. cassinii* displayed defence behaviour, like flipping of wings (in 80% of the experiments; n = 80) and production of disturbance squawks (65%). However, in 55% of all experiments flies managed to stay on the cicada and proceed with infection behaviour.

The third step of the parasitization process, host suitability, was tested in the infection experiments in which a male cicada was presented to acoustically attracted *E. auditrix* (n = 69). During infection a single larva was deposited in the timbal of *M. cassinii*. The larva proceeded into the abdomen of the host (Figure 2B). First instar larvae were found up to five days after infection and second instar larvae started to occur from the third day after infection (Table 1). After six days in captivity most of cicadas were dead, although the tissue damaging due to feeding by the larvae was low. In a surviving cicada, a still second instar larva was found 10 days after infection. In comparison, the fly larvae developed faster in the host cicada, *O. rimosa* (Table 1). Typically, the second stage was reached by day 3 and the third stage was reached by days 5 to 6 post infection. After 6 to 7 days post infection host cicadas were dead caused by the feeding of the fly larva.

**Table 1:** **Larval stage (LS) of** ***E. auditrix*** **in relation to the post infection time in the cicadas** ***M. cassinii*** **and** ***O. rimosa***

**Days post infection**	***M. cassinii***				***O. rimosa***			
	**1. LS (Percent)**	**2. LS (Percent)**	**3. LS (Percent)**	**(n)**	**1. LS (Percent)**	**2. LS (Percent)**	**3. LS (Percent)**	**(n)**
1	100	0	0	4	100	0	0	5
2	100	0	0	6	90	10	0	10
3	88	12	0	8	0	100	0	9
4	50	50	0	8	8	58	33	12
5	50	50	0	2	0	36	64	14
6	0	100	0	3	9	0	91	11
10	0	100	0	1	-	-	-	-

A total of 69 *M. cassinii* and 108 of *O. rimosa* was infected and samples were analysed for larval development each day post infection*.* The larval stage is presented as percent of the individuals in the sample (columns “n”). Note that although in *M. cassinii* no larva reached the third instar only a few cicadas survived more than six days. In *O. rimosa* the third larval instar of *E. auditrix* kills its host after 6 to 7 days.

These results show that *E. auditrix* is able to infect the non-host periodical cicada *M. cassinii* by using its unique acoustic host finding. However, the larvae do not develop completely in the host.

## Discussion

### Host range of *E. auditrix* and parasitization of *M. cassinii*

The data show that in an experimental situation *E. auditrix* is able to locate, accept and infect the periodical cicada *M. cassinii*. It is not known whether *E. auditrix* has a geographical overlap with *M. cassinii.* In the geographical range of *E. auditrix* in Northern Michigan mainly two other cicada species besides *O. rimosa* occur, but no phonotactical attraction to songs of *O. canadensis* (Provancher) or *Tibicen canicularis* (Harris) has been found in field experiments [6]. By contrast, other *Emblemasoma* species prefer calling songs from one species, but can be attracted to other songs as well [11]. Nevertheless in comparison to acoustically orientating Ormiini, species of *Emblemasoma* might have a narrower host range [12]. The host range might be determined by acoustic parameters of the host’s calling songs.

The hearing range of the fly allows listening to the calling songs of a number of cicada species as their frequency spectra often peak between 5 and 10 kHz [13]. The hearing threshold of *E. auditrix* matches well with the spectrum of the song from *M. cassinii*. The mismatch to the spectrum of the host *O. rimosa* is even more surprising and could not be explained so far [6]. Besides frequency content, the temporal structures of the sounds are important for recognition in insects [14]. The parasitoid is adapted to the signal/pulse ratio of the host *O. rimosa* and other patterns are less attractive [6]. Therefore it is not surprising, that the fly has not been attracted to songs of periodical cicadas. However, broadcasting of a song model with the pulse structure of the buzz part from the calling song of *M. cassinii* attracted flies, although the behavioural response was weaker than to the song of the host species.

While the acoustic cue might be a good filter for host selection, what about host infection and host suitability? The host infection behaviour involves a sequence of visual and tactile cues, and is highly specialized with injection of a larva into the timbal of the male cicada [9]. *Emblemasoma auditrix* can apply this whole behavioural sequence to *M. cassinii* although this cicada exhibits more defensive behaviour than *O. rimosa. Emblemasoma auditrix* succeeds in an injection of a larva into the timbal, whereby timbal ribs at a similar location are broken as in *O. rimosa* [9]. Thus, host infection behaviour seems not to be decisive for host selection. However, *M. cassinii* seems to be less suited as host. In *M. cassinii* the larva showed a delayed development (up to five days for the first instar larva) and no larva reached the third stage. The larval development in *O. rimosa* is faster (6 days until emergence of the third instar larva from the host) and has higher survival rates (de Vries & Lakes-Harlan unpublished results). Although the overall mortality of the *M. cassinii* might influence the parasitoid’s larval development, the infection process indicates less suited internal conditions in the periodical cicada. Host selection and host suitability depend on numerous factors [15] and it remains to be shown, whether active defence mechanisms of the cicada or other conditions are responsible for suppressing parasitoid development. Cicada species that are more closely related to *O. rimosa* might be more suited as an (experimental) host for *E. auditrix*.

In summary, a selective response of *E. auditrix* to the acoustic parameters seems to be important, because host infection is unselective and larvae die in non-suited hosts.

### Hypothetical impact of a parasitoid on evolution of periodicity

*M. cassinii* has also been chosen here as an experimental species in respect to the question of evolution of periodicity in cicadas. Models have shown that due to interaction of multiple factors, populations can become periodic or generate outbreaks with huge numbers of individuals [4,16], like the periodical cicadas. One of these factors could be a parasitoid, like *E. auditrix*, which is in the described experimental situation able to infect the periodical cicada *M. cassinii*. Such a scenario is hypothetical but might not be too farfetched, as some lines of arguments can support it. 1) An acoustically hunting parasitoid might explain the low frequency peak of *M. septendecim.* In cicadas a correlation between body length and carrier frequency has been found [17]. *Magicicada cassinii* and *M. septendecula* have carrier frequencies corresponding to their body length. The species *M. septendecim* has a much lower frequency, which is mechanically a result of a reduction of the stiffness of the timbal [18]. The large difference between the peak frequencies of *M. cassinii* and *M. septendecim* does not seem to be necessary for pair-formation, because in 13-year periodical cicadas a difference of 300 Hz is sufficient for species separation [19]. Speculatively, both the lower frequency and the reduced stiffness might be a result of natural selection pressure by a parasitoid like *E. auditrix*. In another system with an acoustically hunting tachinid parasitoid and a calling bushcricket, a low carrier frequency in the calling song has also been attributed to the parasitoid pressure [20]. The reduced stiffness or increased flexibility of the timbal might be advantageous for avoiding parasitization, as in *O. rimosa* [9] as well as in *M. cassinii* usually one or more of the stiff timbal ribs were broken during infection. Soft tissue might be less suited for larvae injection and infection experiments with *M. septendecim* should verify this hypothesis. Thus, both factors might contribute to avoidance of acoustically hunting parasitoids in this species. 2) Infection rates of *O. rimosa* can reach more than 80% in local populations (see below). Together with pathogenic fungi [21,22] and limited environmental carrying capacities [23] these factors might contribute for the evolution of strong periodicity. 3) A hypothetical switch of host species might explain the mismatch of the hearing threshold and the frequency spectrum in the recent host-parasitoid system. In other *Emblemasoma* a match of both, threshold and spectrum, has been reported [11].

Assuming an acoustically hunting parasitoid in the evolution of periodical cicadas, it might have been involved in two different phases. The evolution of the periodical life cycle in the genus *Magicicada* must have preceded species formation [24]. If an acoustically hunting parasitoid was involved in the evolution of periodicity, the selection pressure must have persisted until species formation of *M. septendecim* with its low frequency calling song. Thereafter the parasitoid might have become extinct or, perhaps, even acquired a new host species, like *O. rimosa.* An indication might be the geographic distribution, whereby *M. septendecim* and *O. rimosa* are predominantly found in the Northeast of the US [3], even though today their distribution overlaps only partially.

### Balance in the recent host-parasitoid system

Assuming that *E. auditrix* or an extinct relative could have been a decisive factor for evolution of periodical cicadas and changes in the calling song structure, what effect does the parasitoid impose on the recent host *O. rimosa*? An analysis of the host-parasitoid interactions reveals specific adaptations indicating a long evolutionary trait between both species. The parasitoid has specific sensory and behavioural adaptations (see also above), like the tuning to the temporal and spectral parameters of the calling song [25], the specialized infection behaviour with partial destruction of the sound producing organ [9] and the low number of larvae per parasitoid [26]. The specific adaptations of the host are less obvious. Due to the seemingly very limited external and internal defence capabilities of *O. rimosa* [9], avoidance of parasitization could be a line of defence. This avoidance involves changes in the calling song (as suggested for *M. septendecim*) or changes in the life cycle. Until now no population of *O. rimosa* without parasitoids is known and therefore the effect of the parasitization on the calling song could not be studied yet. An adaptation of the host might be that their populations show large fluctuations in densities, which have been called proto-periodic [1]. However, despite the probable 8–9 year life-cycle [1], adults of *O. rimosa* emerge in each year. Very low numbers in some years might contribute to local breakdowns of parasitoid populations (unpublished data), which, however, might reinvade habitats in years with higher host abundance. On the other hand, a less synchronized emergence of *O. rimosa* (in comparison to the periodical cicadas) and a corresponding slow increase in singing probability might deplete the parasitoid of its larvae and ensure reproduction of the cicada. By the time singing activity peaks, only a few larvae are present in a fly [26] because most larvae are injected into cicadas directly after singing started. This temporal sequence also indicates the high effectiveness of host localisation for an acoustically hunting parasitoid.

## Conclusion

The host range of *E. auditrix* is mainly determined by acoustic parameters. This filter is important, as other sensory cues seem not to be involved in the host selection process and larva will not develop in an unsuited host. Although the recent parasitoid-host system seems to be stable in terms of coexistence of both species, an acoustically hunting parasitoid could have been a selective force during evolution of prime numbered periodicity in cicadas. In our hypothetical scenario combined selective forces might have led to the evolution of proto-periodicity and periodicity. The hypothesis could also explain why only a few species of cicadas became strictly periodic: the sensory mechanism of acoustic host location is rather unique by itself.

## Methods

Recordings of calling songs took place at several locations in Defiance County, Ohio (*Magicicada cassinii*) and in the County of Cincinnati, Ohio (*M. septendecim*). The calling songs were recorded on a DAT recorder (Sony 5 DJA; 44.1 kHz sampling rate) using a Bruel & Kjaer 2203 sound level meter with calibrated microphone. 25 recordings were made from *M. cassinii* and 10 recordings from *M. septendecim* at different locations and in different distances to the calling males. The recordings were compared to those deposited on a webpage of the University of Michigan (http://insects.ummz.lsa.umich.edu/fauna/Michigan_Cicadas/Periodical/Index.html). No differences were found between our recordings and therefore the song models (see below) have been designed according to the temporal structures of calling songs from the webpage. Three songs from single individuals of each species were analyzed by Fast Fourier Transformation Analysis (Hewlett-Packard 5327; 2048 lines; Hanning filter; 44.1 kHz sampling rate).

*Magicicada cassinii* was collected in Defiance County, Ohio (41.184767, −84.311673); the parasitoid fly *Emblemasoma auditrix* and the cicada *O. rimosa* were collected in Emmet County, MI (45.582601, −84.743735). For collection, female flies were attracted to a loudspeaker broadcasting the calling song of the host *O. rimosa* (for details see [6]). Therefore all tested flies were phonotactically active, although their individual age was unknown. The cicadas were kept in screened tents places above some bushes in the field.

Phonotaxis tests in the field (Emmet County, MI) were performed with recorded calling songs of *O. rimosa, M. cassinii* and *M. septendecim* as well as song models for tests with higher sound pressure levels. The song model of *O. rimosa* had a carrier frequency of 9 kHz and a temporal structure with 6 ms pulse duration followed by 6 ms pause; for the song model of *M. cassinii* the buzz part of the calling song [2] was selected due to its temporal structure. The song model had a carrier frequency of 6 kHz and a temporal structure with 3 ms pulse duration followed by a 1 ms pause. Song models from other parts of calling songs form periodical cicadas were not attractive (data not shown). The signals were generated using the CoolEdit software, stored on a compact disc and broadcasted for three minutes each. The sound intensity was adjusted to values between 98 and 100 dB SPL (relative to P_0_ = 2 × 10^−5^ N m^−2^) at 1 m using a sound level meter (Bruel & Kjaer 2203).

The behavioural tests took place in the laboratory at the University of Michigan Biological Station. Therefore flies were kept in small cages with sugar and water *ad libitum*. A fly which had its wings clipped off (n = 22) was released at a starting point in 50 cm distance to a hidden loudspeaker placed in one wall of the test arena (for details see [25]. The signals (as described above) were broadcasted with 80 dB SPL measured at the starting point. The walking behaviour and phonotaxis of the fly was videotaped.

To test host infection, a male *M. cassinii* was attached on top of a loudspeaker broadcasting the calling song of *O. rimosa* to attract flies in the field. After phonotaxis of a fly the sound was turned off and the parasitoid could interact with the potential host. After the fly left the cicada, the latter was analysed for injuries due to the infection. For host suitability experiments, male *M. cassinii* were presented to the parasitoid by holding it at the head on top of the loudspeaker. Such presentation allows infection behaviour by the parasitoid. Infected cicadas (n = 69) were kept in a screened tent placed above some bushes in the field. Samples of infected cicadas were dissected daily and analysed for presence and stage of larvae. After six days in captivity most cicadas were dead; this rate of mortality was the same for infected and non-infected cicadas. Additionally, 108 host cicadas (*O. rimosa*) were infected and kept under the same conditions as *M. cassinii* above. Photos were taken on a stereo microscope equipped with a CCD camera.
